# Association of Heavy Alcohol Intake and *ALDH2* rs671 Polymorphism With Hepatocellular Carcinoma and Mortality in Patients With Hepatitis B Virus–Related Cirrhosis

**DOI:** 10.1001/jamanetworkopen.2022.23511

**Published:** 2022-07-25

**Authors:** Ming-Chao Tsai, Sien-Sing Yang, Chih-Che Lin, Wen-Lun Wang, Yao-Chun Hsu, Yaw-Sen Chen, Jui-Ting Hu, James Yu Lin, Ming-Lung Yu, Chih-Wen Lin

**Affiliations:** 1 Division of Hepato-Gastroenterology, Department of Medicine, Kaohsiung Chang Gung Memorial Hospital, Chang Gung University College of Medicine, Kaohsiung, Taiwan; 2 Liver Unit, Cathay General Hospital, Taipei, Taiwan; 3 Department of Surgery, Kaohsiung Chang Gung Memorial Hospital, Chang Gung University College of Medicine, Kaohsiung, Taiwan; 4 Division of Gastroenterology and Hepatology, Department of Medicine, E-Da Hospital, I-Shou University, Kaohsiung, Taiwan; 5 School of Medicine, College of Medicine, I-Shou University, Kaohsiung, Taiwan; 6 Department of Surgery, E-Da Hospital, I-Shou University, Kaohsiung, Taiwan; 7 Kaohsiung American School, Kaohsiung, Taiwan; 8 Hepatobiliary Section, Department of Internal Medicine, Hepatitis Center, Kaohsiung Medical University Hospital, Kaohsiung, Taiwan; 9 School of Medicine and Hepatitis Research Center, College of Medicine, Center for Liquid Biopsy and Cohort Research, Kaohsiung Medical University, Kaohsiung, Taiwan; 10 Division of Gastroenterology and Hepatology, E-Da Dachang Hospital, I-Shou University, Kaohsiung, Taiwan; 11 School of Chinese Medicine, College of Chinese Medicine, Research Center for Traditional Chinese Medicine, China Medical University, Taichung, Taiwan

## Abstract

**Importance:**

The role of heavy alcohol intake, aldehyde dehydrogenase 2 gene (*ALDH2*) rs671 polymorphism, and hepatitis B virus (HBV) infection in hepatocellular carcinoma (HCC) development and mortality remains uncertain.

**Objective:**

To investigate the association of heavy alcohol intake, *ALDH2* rs671 polymorphism, and HBV infection with HCC development and mortality in patients with cirrhosis.

**Design, Setting, and Participants:**

This retrospective cohort study enrolled patients with cirrhosis with heavy alcoholism or/and HBV infection from January 2005 to December 2020. Patients were followed up through June 30, 2021. The current data analysis was performed from August 2021 to April 2022. Patients from 3 tertiary hospitals in Taiwan were enrolled.

**Exposures:**

Heavy alcohol intake was defined as consuming more than 80 g of ethanol each day for at least 5 years.

**Main Outcomes and Measures:**

The primary end point was newly developed HCC. The secondary end point was overall mortality.

**Results:**

Of 1515 patients with cirrhosis (342 with concomitant heavy alcoholism and HBV infection, 796 with HBV infection alone, and 377 with heavy alcoholism alone), 1277 (84.3%) were men, and their mean (SD) age was 49.5 (10.2) years; 746 patients had blood samples collected for *ALDH2* rs671 polymorphism analysis. The 10-year cumulative incidences of HCC and mortality were significantly higher in patients with cirrhosis with concomitant HBV infection and alcoholism than in those with HBV infection alone or alcoholism alone. Heavy alcohol intake and the *ALDH2* rs671 genotype (GA/AA) were associated with significantly increased risk of HCC and mortality in patients with HBV-related cirrhosis. In patients with cirrhosis with concomitant HBV infection and alcoholism, factors associated with risk of HCC were baseline serum HBV DNA (adjusted hazard ratio [aHR], 3.24; 95% CI, 1.43-7.31), antiviral therapy (aHR, 0.15; 95% CI, 0.05-0.39), alcohol intake (aHR, 1.78; 95% CI, 1.02-3.12), abstinence (aHR, 0.32; 95% CI, 0.18-0.59), and *ALDH2* rs671 polymorphism (aHR, 5.61; 95% CI, 2.42-12.90). Factors associated with increased risk of mortality were abstinence (aHR, 0.25; 95% CI, 0.16-0.32), *ALDH2* rs671 polymorphism (aHR, 1.58; 95% CI, 1.09-2.26), Child-Pugh class B vs A (aHR, 1.43; 95% CI, 1.13-2.25) and class C vs A (aHR, 1.98; 95% CI, 1.18-3.31), serum albumin (aHR, 0.61; 95% CI, 0.43-0.86), and HCC development (aHR, 1.68; 95% CI, 1.12-2.89).

**Conclusions and Relevance:**

These findings suggest that heavy alcohol intake and *ALDH2* rs671 polymorphism are associated with significantly increased risk of HCC development and mortality in patients with HBV-related cirrhosis. Patients with these risk factors should be monitored closely for HCC.

## Key Points

**Question:**

What is the association of heavy alcohol intake, aldehyde dehydrogenase 2 gene (*ALDH2*) rs671 polymorphism, and hepatitis B virus (HBV) infection with hepatocellular carcinoma (HCC) development and mortality in patients with cirrhosis?

**Findings:**

In this cohort study of 1515 patients with cirrhosis, the 10-year cumulative incidences of HCC and mortality were significantly higher in patients with concomitant HBV infection and alcoholism than in those with HBV infection alone or alcoholism alone. Heavy alcohol intake and the *ALDH2* rs671 genotype (GA/AA) were associated with significantly increased incidence and risk of HCC and mortality in patients with HBV-related cirrhosis.

**Meaning:**

These findings suggest that heavy alcohol intake and *ALDH2* rs671 polymorphism are associated with significantly increased risk of HCC development and mortality in patients with HBV-related cirrhosis, and patients with these risk factors should be monitored closely for HCC.

## Teaser

This cohort study investigates the association of heavy alcohol intake, aldehyde dehydrogenase 2 gene (*ALDH2*) rs671 polymorphism, and hepatitis B virus infection with hepatocellular carcinoma development and mortality in patients with cirrhosis.

## Introduction

Hepatocellular carcinoma (HCC) is the fifth most commonly occurring cancer and the second most common cause of cancer-related death worldwide.^1,2,3,4,5^ Daily alcohol intake of more than 80 g for at least 5 years has been shown to enhance the progression to cirrhosis and the development of HCC and to increase mortality in Western countries and Eastern Asia.^6,7,8,9^ Hepatitis B virus (HBV) infection with elevated serum HBV DNA levels has been defined as an important factor associated with risk of cirrhosis, HCC, and mortality.^10,11^ Antiviral therapy has been widely used to reduce the development of HCC and mortality in patients with HBV and fibrosis or cirrhosis.^12,13,14,15^ The synergistic effect of alcohol intake and HBV infection on the development of HCC has been reported.^7,16,17^ Furthermore, our previous study^8^ demonstrated that heavy alcohol consumption was associated with increased incidence of HCC in patients with HBV-related cirrhosis and that antiviral therapy was associated with reduced risk of HCC in patients with cirrhosis with HBV infection and alcoholism. In addition, the aldehyde dehydrogenase 2 gene (*ALDH2*) polymorphism affects the development of HCC in patients with alcoholism with or without viral hepatitis^18,19,20,21^ and in patients without alcoholism.^22,23^ Some studies^24,25,26,27^ have demonstrated that the *ALDH2* rs671 polymorphism is not associated with HCC in East Asian individuals. However, the roles of heavy alcohol intake, *ALDH2* rs671 polymorphism, and HBV infection in the development of HCC and mortality remain uncertain and need to be explored. Therefore, we investigated the association of heavy alcohol intake, *ALDH2* rs671 polymorphism, and HBV infection with HCC development and mortality in patients with cirrhosis. We also explored the factors associated with increased risk of HCC and mortality in patients with cirrhosis with concomitant HBV infection and heavy alcoholism.

## Methods

### Study Design and Patients

This cohort study was approved by the ethics committee of each participating institution and was conducted in accordance with the ethical guidelines of the Declaration of Helsinki.^28^ The patients provided signed informed consent forms for study participation. The report of this study followed the Strengthening the Reporting of Observational Studies in Epidemiology (STROBE) reporting guideline for cohort studies.

We retrospectively enrolled patients with cirrhosis at the E-DA Hospital/I-SHOU University, Kaohsiung, South Taiwan, Kaohsiung Chang Gung Memorial Hospital, South Taiwan, and Cathay General Hospital, Taipei, North Taiwan, from January 1, 2005, to December 31, 2020. The inclusion criteria of the participants were patients with cirrhosis with heavy alcoholism (defined as >80 g of ethanol each day for at least 5 years) and/or HBV infection (serum hepatitis B surface antigen positivity for more than 6 months). The exclusion criteria consisted of the presence of hepatitis C virus infection, alcohol intake of less than 80 g per day and for less than 5 years, patients with HCC at enrollment, and incomplete data (ie, we could not find the events or date on HCC and mortality) (eFigure 1 in the Supplement). All patients were monitored for more than 6 months. We followed these patients until June 30, 2021. Liver cirrhosis was clinically defined according to histologically confirmed cirrhosis. Imaging findings, including ultrasonography, acoustic radiation force impulse, FibroScan, computed tomography, and magnetic resonance imaging, were evaluated to confirm the presence of liver cirrhosis, or endoscopy was used to confirm the presence of varices.

### Outcome Ascertainment

The primary end point was newly developed HCC, and the secondary end point was overall mortality after at least 6 months of follow-up. All patients underwent α-fetoprotein tests and imaging examinations, including ultrasonography, computed tomography, or magnetic resonance imaging, every 3 to 6 months or as necessary for HCC screening. HCC was diagnosed on the basis of the results of histological examination or typical HCC imaging findings according to the American Association for the Study of Liver Diseases HCC guidelines.^3^ Cases of newly developed HCC cases and related mortality outcomes were ascertained from the medical records (1237 patients [81.7%]) of the enrolled centers or from National Cancer Registry data according to the *International Classification of Disease, Ninth Revision* (278 patients [18.3%]) for patients who were lost to follow-up in the enrolled centers. The factors associated with risk of HCC and mortality were analyzed in patients with concomitant heavy alcoholism and HBV infection. The follow-up time was defined as the time from the date of inclusion to the date of death, the last follow-up, or the end of the study (June 30, 2021), whichever came first, and the occurrence time was defined as the time from the date of inclusion to the date of HCC diagnosis, the date of death, the last follow-up, or the end of the study (June 30, 2021).

### Alcoholism

Patients with alcoholism were encouraged to abstain from alcohol. Abstinence was defined as abstaining from alcohol for more than 6 months during follow-up.

### *ALDH2* rs671 Polymorphism

*ALDH2* is a major enzyme for acetaldehyde elimination. The ALDH2*2 allele variant is a single point variation (G to A) in exon 12, resulting in a change from glutamine to lysine at codon 487 and the inactivation of *ALDH2* enzyme activity in humans, causing deficiency. The presence of a single polymorphism (glutamine to lysine, G to A, or *1 to *2) was evaluated in the blood samples. The *ALDH2* rs671 polymorphism causes 1 of 3 genotypes: GG, AA, and GA. We combined patients with the GA and AA genotypes into a single GA/AA group for *ALDH2* deficiency.

### Statistical Analysis

Analysis of the long-term data was performed in August 2020. The current data analysis was performed from August 2021 to April 2022. Continuous data are expressed as the mean (SD). Categorical data are described using numbers and percentages. Normally distributed continuous variables were compared by *t* test or 1-way analysis of variance, and the Wilcoxon rank-sum test was applied for comparisons of 2 groups when continuous variables were not normally distributed. The χ^2^ test was used to compare categorical variables. Observations with missing data (incomplete data) were regarded as random occurrences and were not included in the analyses. The cumulative incidence of newly developed HCC and mortality were evaluated using the Kaplan-Meier method. Moreover, we used logistic regression to perform propensity score matching (PSM) with sex, age, body mass index, and Child-Pugh class to reduce selection bias in our analyses (eTables 1-3 in the Supplement). Each group was matched with the control group according to the generated propensity scores using a caliper width of 0.02. A standardized mean difference of less than 20% was used to analyze the covariate balance after PSM. Because patients who died were no longer at risk for HCC development, competing risk analyses were conducted to evaluate the cumulative incidence of newly developed HCC, with mortality considered a competing risk. The factors associated with risk of HCC and mortality were evaluated by univariable and multivariable analyses. Multivariable analyses were performed with Cox proportional regression modes for HCC after adjusting for body mass index, baseline HBV DNA, antiviral therapy, alcohol intake amount, abstinence, *ALDH2* rs671 genotype, alanine aminotransferase, γ-glutamyltransferase, and α-fetoprotein; and for mortality after adjusting for hepatitis B e antigen, baseline HBV DNA, antiviral therapy, alcohol intake amount, abstinence, *ALDH2* rs671 genotype, Child-Pugh class, albumin, and newly developed HCC. Two-sided *P* < .05 was considered statistically significant. All analyses were performed using SPSS statistical software version 23.0 (IBM). Study definitions can be found in eAppendix 1 in the Supplement.

## Results

### Baseline Demographic Characteristics

A total of 5168 patients were enrolled retrospectively. After exclusions, 1515 patients remained. The demographic features of the 1515 patients with cirrhosis are shown in Table 1. Most patients were men (1277 patients [84.3%]), and their mean (SD) age was 49.5 (10.2) years. Of the 1515 patients with cirrhosis, 342 (22.6%) had concomitant HBV infection and alcoholism (mean [SD] age, 46.2 [9.3] years; mean [SD] follow-up, 4.0 [3.2] years), 796 (52.5%) had HBV infection alone (mean [SD] age, 50.5 [10.4] years; mean [SD] follow-up, 4.7 [3.1] years), and 377 (24.9%) had alcoholism alone (mean [SD] age, 50.6 [9.9] years; mean [SD] follow-up, 5.1 [3.5] years).

**Table 1.** Demographic Data of Patients With Cirrhosis

Characteristics	Patients, No. (%)				
	Total (N = 1515)	HBV plus alcoholism (n = 342)	HBV only (n = 796)	Alcoholism only (n = 377)	*P* value^a^
Age, mean (SD), y	49.5 (10.2)	46.2 (9.3)^b^	50.5 (10.4)	50.6 (9.9)^c^	<.001
Sex					
Male	1277 (84.3)	314 (91.8)^b^	647 (81.3)	316 (83.8)^c^	<.001
Female	238 (15.7)	28 (8.2)^b^	149 (18.7)	61 (16.2)^c^	
Body mass index^d^	24.0 (3.6)	24.9 (3.6)^b^	23.7 (3.4)	23.8 (3.9)^c^	<.001
Alcohol intake, mean (SD), g/d	86 (160)	186 (81)^b^	0^e^	173 (86)^c^	<.001
Alcohol intake duration, mean (SD), y	8.4 (9.9)	18.2 (6.6)^b^	0^e^	17.4 (6.2)	<.001
Abstinence	408 (56.7)	171 (50)	NA	237 (62.9)^c^	<.001
Aspartate aminotransferase, mean (SD), U/L	144 (127)	120 (143)^b^	152 (121)	148 (120)^c^	<.001
Alanine aminotransferase, mean (SD), U/L	63 (55)	63 (49)	64 (58)	61 (53)	.71
Total bilirubin, mean (SD), mg/dL	3.6 (5.1)	2.7 (2.8)^b^	4.0 (5.6)	3.8 (5.2)^c^	<.001
Alkaline phosphatase, mean (SD), IU/L	347 (202)	384 (236)^b^	322 (180)^e^	366 (203)^c^	<.001
γ-Glutamyltransferase, mean (SD), IU/L	328 (303)	279 (309)	331 (303)	348 (299)^c^	.56
Albumin, mean (SD), g/dL	3.3 (0.6)	3.3 (0.6)	3.3 (0.6)	3.3 (0.6)	.88
Platelet count, mean (SD), ×10^3^/mL	109 (102)	75 (66)^b^	120 (106)	116 (113)^c^	<.001
International normalized ratio, mean (SD)	1.3 (0.3)	1.3 (0.5)	1.3 (0.3)	1.3 (0.3)	.63
α-Fetoprotein, mean (SD), ng/mL	29 (98)	39 (117)	27 (95)	25 (86)	.09
Hepatitis B surface antigen positive	1138 (75.1)	342 (100)	796 (100)^e^	0^c^	<.001
Hepatitis B e antigen positive	360 (23.8)	90 (26.3)^b^	270 (33.9)	NA	<.001
Baseline HBV DNA, mean (SD), log_10_ IU/mL	3.2 (2.7)	4.3 (2.1)	4.2 (2.3)	NA	.12
Baseline HBV DNA ≥5 log_10_ IU/mL	449 (29.6)	136 (39.8)	313 (39.3)	NA	.65
Antiviral viral therapy positive	975 (64.5)	308 (90.1)^b^	667 (83.8)	NA	<.001
Child-Pugh class					
A	644 (42.5)	172 (50.2)^b^	317 (39.8)	155 (41.1)^c^	<.001
B	553 (36.5)	85 (24.9)^b^	316 (39.7)	152 (40.3)^c^	<.001
C	318 (21)	85 (24.9)^b^	163 (20.5)	70 (18.6)^c^	<.001
Follow-up time, mean (SD), y	4.6 (3.3)	4.0 (3.2)^b^	4.7 (3.1)	5.1 (3.5)^c^	<.001
Newly developed HCC	270 (17.8)	81 (23.7)^b^	134 (16.8)	55 (14.6)^c^	.004
Annual HCC incidence rate, %/y	3.5	5.9^b^	3.6	2.9^c^	<.001
Mortality	627 (41.4)	155 (45.3)	322 (40.5)	150 (39.8)	.23
Annual mortality incidence rate, %/y	8.3	11.3^b^	8.6	7.9^c^	<.001

Abbreviations: HBV, hepatitis B virus; HCC, hepatocellular carcinoma; NA, data not available.

SI conversion factors: To convert α-fetoprotein to micrograms per liter, multiply by 1; γ-glutamyltransferase to microkatals per liter, multiply by 0.0167; alanine aminotransferase to microkatals per liter, multiply by 0.0167; albumin to grams per liter, multiply by 10; alkaline phosphatase to microkatals per liter, multiply by 0.0167; aspartate aminotransferase to microkatals per liter, multiply by 0.0167; bilirubin to micromoles per liter, multiply by 17.104; platelet count, to 10^9^ per liter, multiply by 1.

^a^ *P* value is calculated by 1-way analysis of variance test among 3 groups.

^b^ *P* < .05, HBV and alcoholism vs HBV.

^c^ *P* < .05, HBV vs alcoholism; *P* value is calculated by *t* tests, Wilcoxon rank-sum statistics, or χ^2^ tests.

^d^ Body mass index is calculated as weight in kilograms divided by height in meters squared.

^e^ *P* < .05, HBV and alcoholism vs alcoholism.

### Newly Developed HCC in All Patients With Cirrhosis

**Figure 1.**
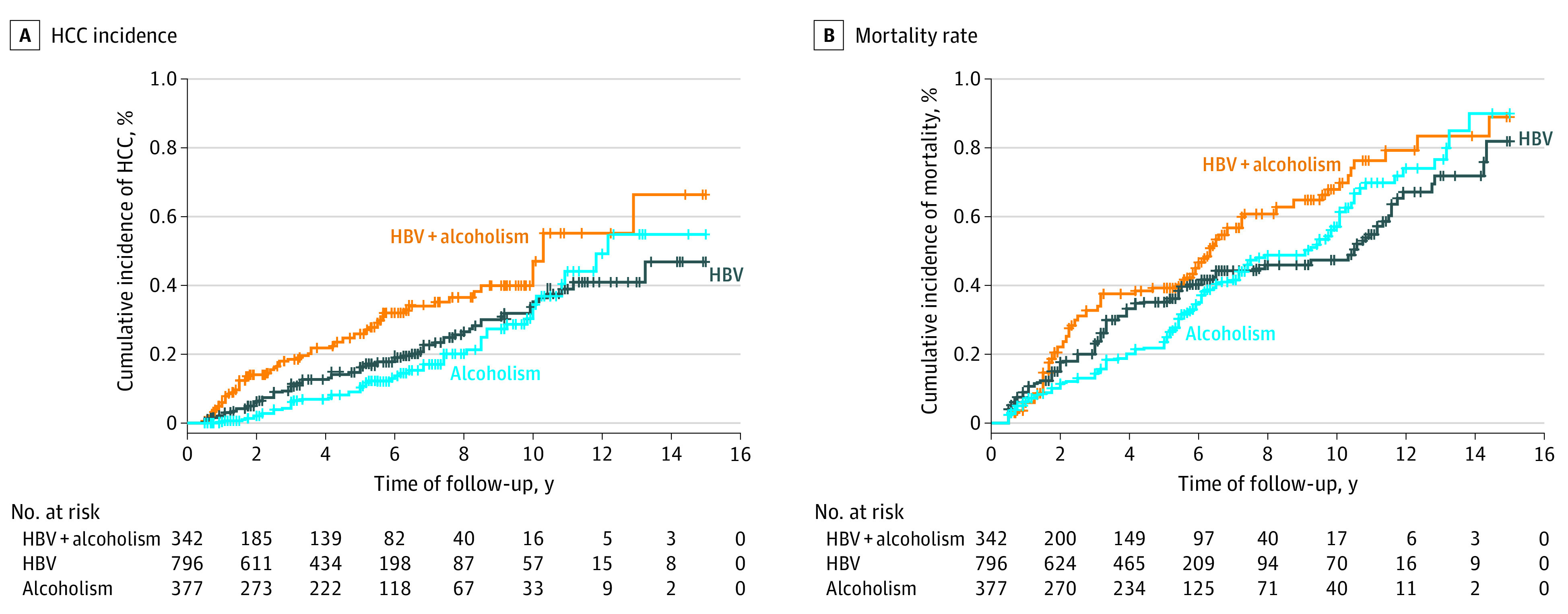
Cumulative Incidence of Hepatocellular Carcinoma (HCC) and Mortality in All Patients The cumulative incidences of HCC (A) and mortality (B) were higher in all patients with cirrhosis with concomitant hepatitis B virus (HBV) infection and alcoholism than in those with HBV infection alone or alcoholism alone. Vertical lines denote data censoring.

The cumulative incidence rates of HCC in these groups were 6.0% at 1 year, 25.9% at 5 years, and 47.2% at 10 years in patients with concomitant HBV infection and alcoholism; 2.2% at 1 year, 16.2% at 5 years, and 35.2% at 10 years in patients with HBV infection alone; and 0.3% at 1 year, 10.3% at 5 years, and 34.4% at 10 years in patients with alcoholism alone (Figure 1A). The 10-year cumulative incidence rates of HCC were higher in patients with concomitant HBV infection and alcoholism than in those with HBV infection alone (crude hazard ratio [HR], 1.74; 95% CI, 1.31-2.29; *P* < .001) or with alcoholism alone (crude HR, 2.16; 95% CI, 1.53-3.04; *P* < .001), as shown in Figure 1A.

### Mortality in All Patients With Cirrhosis

The cumulative mortality rates were 5.9% at 1 year, 29.3% at 5 years, and 67.9% at 10 years for patients with concomitant HBV infection and alcoholism; 9.0% at 1 year, 35.1% at 5 years, and 47.4% at 10 years for patients with HBV infection alone; and 6.3% at 1 year, 23.6% at 5 years, and 57.1% at 10 years for patients with alcoholism alone (Figure 1B). The 10-year cumulative mortality rates were higher in patients with concomitant HBV infection and alcoholism than in those with HBV infection alone (crude HR, 1.85; 95% CI, 1.53-2.25; *P* < .001) and those with alcoholism alone (crude HR, 1.60; 95% CI, 1.27-2.01; *P* < .001), as shown in Figure 1B.

### Newly Developed HCC and Mortality After PSM

Regarding newly developed HCC, after PSM, patients with concomitant HBV infection and alcoholism had a significantly higher incidence of newly developed HCC than those with HBV infection alone (crude HR, 2.13; 95% CI, 1.62-2.81; *P* < .001) (eFigure 2A in the Supplement) and those with alcoholism alone (crude HR, 2.27; 95% CI, 1.52-3.35; *P* < .001) (eFigure 2B in the Supplement). In the competing risk analysis, patients with concomitant HBV infection and alcoholism still had a significantly higher incidence of HCC than those with HBV infection alone (crude HR, 2.66; 95% CI, 1.99-3.46; *P* < .001) (eFigure 3A in the Supplement) and those with alcoholism alone (crude HR, 1.96; 95% CI, 1.39-2.74; *P* < .001) (eFigure 3B in the Supplement). There was no significant difference in the incidence of HCC between patients with HBV infection alone and patients with alcoholism alone after PSM (eFigure 2C in the Supplement) and in the competing risk analysis (eFigure 3C in the Supplement).

Regarding mortality, after PSM, patients with concomitant HBV infection and alcoholism had a significantly higher incidence of mortality than those with HBV infection alone (crude HR, 1.68; 95% CI, 1.39-2.03; *P* < .001) (eFigure 2D in the Supplement) and those with alcoholism alone (crude HR, 1.45; 95% CI, 1.12-1.85; *P* < .001) (eFigure 2E in the Supplement). There was no significant difference in the incidence of mortality between patients with HBV infection alone and patients with alcoholism alone after PSM (eFigure 2F in the Supplement).

### Association of *ALDH2* rs671 Polymorphism With HCC Development and Related Mortality

We prospectively assessed 746 patients with cirrhosis with HBV infection and/or heavy alcoholism for *ALDH2* rs671 polymorphism analysis (Table 2). The incidence of the genotype of GA/AA was higher in patients with HBV infection alone (164 of 245 patients [66.9%]) and alcoholism alone (116 of 207 patients [56.0%]) than those in patients with concomitant HBV infection and alcoholism (137 of 294 patients [44.6%]).

**Table 2.** Association of *ALDH2* rs671 Polymorphism With Newly Developed HCC and Mortality in Patients With Cirrhosis

Characteristic and *ALDH2* rs671 genotype	Patients, No./total No. (%)				*P* value^a^
	Total (N = 746)	HBV plus alcoholism (n = 294)	HBV only (n = 245)	Alcoholism only (n = 207)	
Genotypes					
GG	329/746 (44.1)	157/294 (55.4)^b^	81/245 (33.1)^c^	91/207 (44.0)^d^	<.001
GA/AA	417/746 (55.9)	137/294 (44.6)	164/245 (66.9)	116/207 (56.0)	
Newly developed HCC					
GG	36/329 (10.9)	7/157 (4.5)^b^	23/81 (28.4)^c^	6/91 (6.6)^d^	<.001
GA/AA	147/417 (35.3)	66/137 (48.2)	50/164 (30.5)	31/116 (26.7)	
Crude HR (95% CI)	12.60 (5.80-27.60)	10.50 (4.80-22.80)	1.13 (0.68-1.91)	4.68 (1.94-11.20)	
*P* value^e^	<.001	<.001	.62	.001	NA
Mortality					
GG	109/329 (33.1)	51/157 (42.5)^b^	36/81 (44.4)^c^	22/91 (24.2)^d^	<.001
GA/AA	250/417 (60.0)	91/137 (66.4)	82/164 (50.0)	77/116 (66.4)	
Crude HR (95% CI)	1.46 (1.04-2.05)	1.55 (1.09-2.19)	0.99 (0.67-1.47)	3.01 (1.87-4.86)	
*P* value^e^	.03	.01	.97	<.001	NA

Abbreviations: HCC, hepatocellular carcinoma; HR, hazards ratio; NA, not applicable.

^a^ *P* value is calculated by 1-way analysis of variance test among 3 groups.

^b^ *P* < .05, HBV and alcoholism vs HBV.

^c^ *P* < .05, HBV and alcoholism vs alcoholism.

^d^ *P* < .05, HBV vs alcoholism; *P* value is calculated by χ^2^ tests.

^e^ *P* value is calculated by Cox regression analyses.

The GA/AA genotype was significantly associated with an increased incidence of newly developed HCC compared with the GG genotype in patients with concomitant HBV infection and alcoholism (crude HR, 10.50; 95% CI, 4.80-22.80; *P* < .001) and in those with alcoholism alone (crude HR, 4.68; 95% CI, 1.94-11.20; *P* = .001) but not in those with HBV infection alone (Table 2). The GA/AA genotype was significantly associated with increased mortality compared with the GG genotype in patients with concomitant HBV infection and alcoholism (crude HR, 1.55; 95% CI, 1.09-2.19; *P* = .01) and in those with alcoholism alone (crude HR, 3.01; 95% CI, 1.87-4.86; *P* < .001) but not in those with HBV infection alone (Table 2).

### Factors Associated With Risk of HCC Development and Mortality in Patients With Concomitant HBV Infection and Heavy Alcoholism

**Figure 2.**
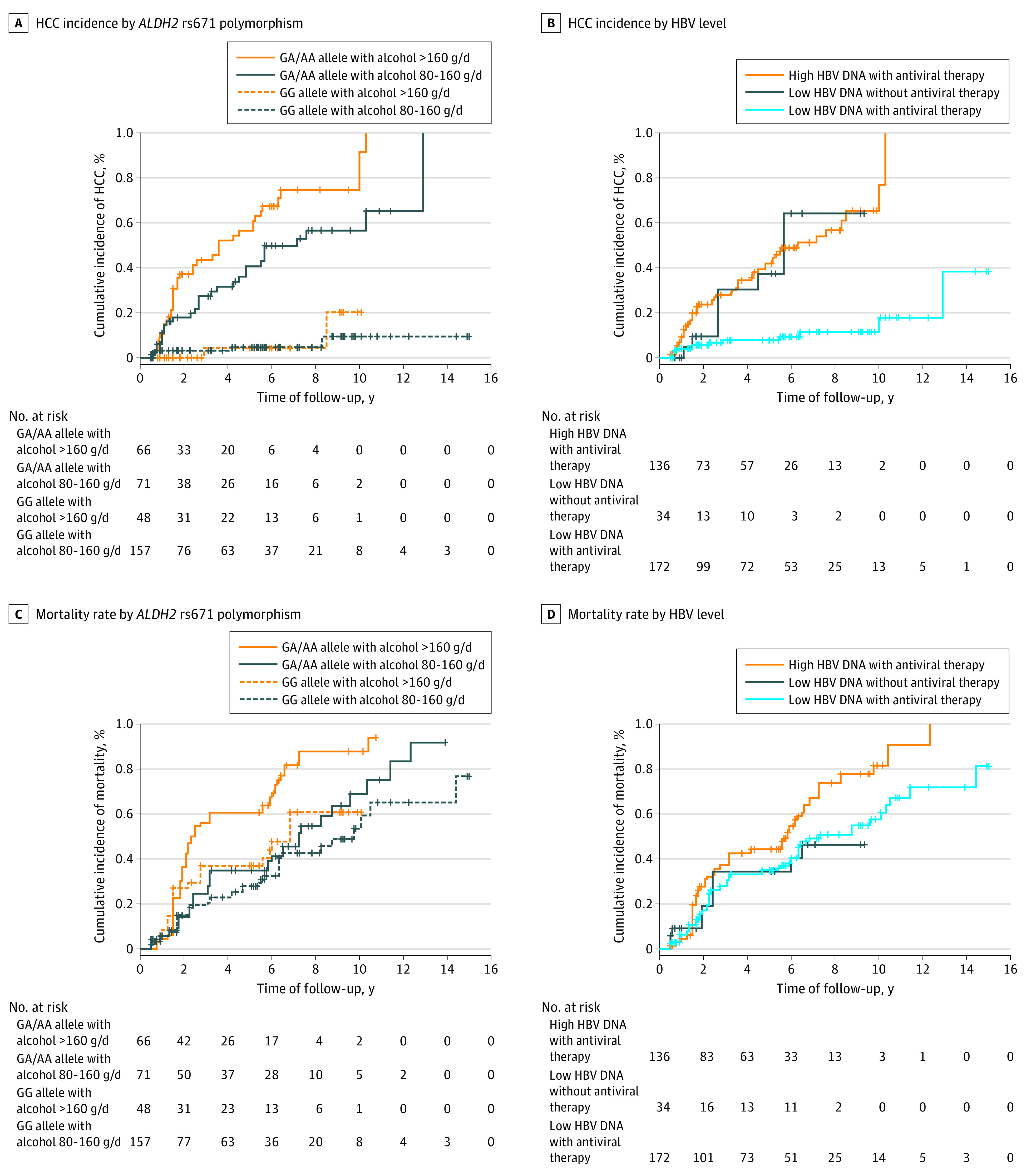
Cumulative Incidences of Hepatocellular Carcinoma (HCC) and Mortality According to Alcohol Intake With *ALDH2* rs671 Polymorphism and Serum Hepatitis B Virus (HBV) DNA Levels and the Administration of Antiviral Therapy The GA/AA genotype with alcohol intake greater than 160 g per day was significantly associated with increased incidences of HCC and mortality compared with the GG genotype with alcohol intake of 80 to 160 g per day (A and C). High serum HBV DNA levels and the administration of antiviral therapy were significantly associated with increased incidences of HCC and mortality compared with low serum HBV DNA levels and the administration of antiviral therapy (B and D). Vertical lines denote data censoring.

In 342 patients with cirrhosis with concomitant HBV infection and heavy alcoholism, for newly developed HCC, baseline serum HBV DNA (≥5 log_10_ IU/mL) (adjusted HR, 3.24; 95% CI, 1.43-7.31; *P* = .005), antiviral therapy (adjusted HR, 0.15; 95% CI, 0.05-0.39; *P* < .001), alcohol intake amount (>160 vs 80-160 g per day; adjusted HR, 1.78; 95% CI, 1.02-3.12; *P* = .04), abstinence (adjusted HR, 0.32; 95% CI, 0.18-0.59; *P* < .001), and *ALDH2* rs671 polymorphism (GA/AA vs GG; adjusted HR, 5.61; 95% CI, 2.42-12.90; *P* < .001) remained significantly associated with the incidence of HCC in the multivariable regression analysis (Table 3). In addition, the presence of the GA/AA genotype and alcohol intake greater than 160 g per day (crude HR, 18.10; 95% CI, 7.62-42.60; *P* < .001) or 80 to 160 g per day (HR, 9.72; 95% CI, 4.04-23.30; *P* < .001) were significantly associated with an increased incidence of HCC compared with the presence of the GG genotype and an alcohol intake 80 to 160 g per day (Figure 2A). High serum HBV DNA levels (≥5 log_10_ IU/mL) and the administration of antiviral therapy (crude HR, 6.58; 95% CI, 3.57-12.10; *P* < .001) or low serum HBV DNA levels (<5 log_10_ IU/mL) and no antiviral therapy (crude HR, 5.69; 95% CI, 2.40-13.50; *P* < .001) were significantly associated with an increased incidence of HCC compared with low serum HBV DNA levels (<5 log_10_ IU/mL) and the administration of antiviral therapy (Figure 2B).

**Table 3.** Univariable and Multivariable Cox Regression Analyses of the Factors Associated With Newly Developed HCC and Mortality in Patients With Cirrhosis With Concomitant HBV Infection and Heavy Alcoholism

Characteristics (N = 342)	Newly developed HCC				Mortality			
	Univariable regression		Multivariable regression		Univariable regression		Multivariable regression	
	Crude HR (95% CI)	*P* value	Adjusted HR (95% CI)	*P* value	Crude HR (95% CI)	*P* value	Adjusted HR (95% CI)	*P* value
Age (>50 vs ≤50 y)	1.23 (0.78-1.85)	.31	NA	NA	1.18 (0.83-1.77)	.34	NA	NA
Sex (male vs female)	0.55 (0.28-1.07)	.08	NA	NA	0.66 (3.95-1.11)	.12	NA	NA
Body mass index (>24 vs ≤24)^a^	0.51 (0.31-0.85)	.02	0.96 (0.87-1.02)	.11	0.91 (0.90-1.08)	.61	NA	NA
Hepatitis B e antigen (positive vs negative)	1.49 (0.94-2.36)	.09	NA	NA	1.62 (1.17-2.26)	.004	1.13 (0.78-1.61)	.45
Baseline HBV DNA (≥5 vs <5 log_10_ IU/mL)	4.28 (2.60-7.04)	<.001	3.24 (1.43-7.31)	.005	1.63 (1.19-2.25)	.002	1.22 (0.80-1.83)	.33
Antiviral therapy (yes vs no)	0.39 (0.21-0.71)	.002	0.15 (0.05-0.39)	<.001	0.58 (0.33-0.92)	.04	0.60 (0.27-1.38)	.14
Alcohol intake amount (>160 vs 80-160 g/d)	1.85 (1.18-2.91)	.002	1.78 (1.02-3.12)	.04	2.12 (1.67-3.27)	<.001	1.35 (0.72-2.39)	.29
Alcohol intake duration (>18 vs ≤18 y)	0.92 (0.89-1.09)	.92	NA	NA	0.91 (0.87-1.15)	.86	NA	NA
Abstinence (yes vs no)	0.24 (0.14-0.39)	<.001	0.32 (0.18-0.59)	<.001	0.22 (0.13-0.40)	<.001	0.25 (0.16-0.32)	<.001
*ALDH2* rs671 genotype (GA/AA vs GG)	10.5 (4.80-22.80)	<.001	5.61 (2.42-12.90)	<.001	1.68 (1.06-2.69)	<.001	1.58 (1.09-2.26)	.02
Child-Pugh class (B vs A)	1.42 (0.85-2.36)	.17	NA	NA	2.43 (1.63-3.62)	<.001	1.43 (1.13-2.25)	.04
Child-Pugh class (C vs A)	0.87 (0.49-1.56)	.65	NA	NA	2.49 (1.68-3.71)	<.001	1.98 (1.18-3.31)	.009
Aspartate aminotransferase (>40 vs ≤40 U/L)	0.94 (0.91-1.07)	.96	NA	NA	0.86 (0.83-1.23)	.41	NA	NA
Alanine aminotransferase (>40 vs ≤40 U/L)	1.71 (1.18-2.85)	.04	1.19 (0.84-1.75)	.39	1.48 (0.69-1.77)	.62	NA	NA
Total bilirubin (>1.5 vs ≤1.5 mg/dL)	1.19 (0.91-1.18)	.90	NA	NA	1.32 (0.73-2.48)	.43	NA	NA
Alkaline phosphatase (>350 vs ≤350 IU/L)	1.36 (0.68-2.65)	.33	NA	NA	1.12 (0.87-1.25)	.73	NA	NA
γ-Glutamyltransferase (>330 vs ≤330 IU/L)	1.98 (1.37-3.26)	.02	1.26 (0.81-1.98)	.26	1.55 (0.52-2.98)	.21	NA	NA
Albumin (>3.5 vs ≤3.5 g/dL)	0.83 (0.43-1.77)	.56	NA	NA	0.46 (0.35-0.60)	<.001	0.61 (0.43-0.86)	.005
Platelet count (150 vs ≤150 ×10^3^/mL)	1.23 (0.86-1.25)	.82	NA	NA	1.46 (0.72-1.71)	.68	NA	NA
International normalized ratio (>1.1 vs ≤1.1)	1.33 (0.69-2.55)	.39	NA	NA	1.88 (0.98-3.23)	.06	NA	NA
α-Fetoprotein (>200 vs ≤200 ng/mL)	1.89 (1.36-3.17)	<.001	1.38 (0.69-2.41)	.15	1.33 (0.70-2.58)	.44	NA	NA
Newly developed HCC (yes vs no)	NA	NA	NA	NA	1.72 (1.32-3.85)	.02	1.68 (1.12-2.89)	.01

Abbreviations: HBV, hepatitis B virus; HCC, hepatocellular carcinoma; HR, hazard ratio; NA, data not available.

SI conversion factors: To convert α-fetoprotein to micrograms per liter, multiply by 1; γ-glutamyltransferase to microkatals per liter, multiply by 0.0167; alanine aminotransferase to microkatals per liter, multiply by 0.0167; albumin to grams per liter, multiply by 10; alkaline phosphatase to microkatals per liter, multiply by 0.0167; aspartate aminotransferase to microkatals per liter, multiply by 0.0167; bilirubin to micromoles per liter, multiply by 17.104; platelet count, to 10^9^ per liter, multiply by 1.

^a^ Body mass index is calculated as weight in kilograms divided by height in meters squared.

Regarding mortality, in the multivariable regression analysis, abstinence (adjusted HR, 0.25; 95% CI, 0.16-0.32; *P* < .001), *ALDH2* rs671 polymorphism (GA/AA vs GG; adjusted HR, 1.58; 95% CI, 1.09-2.26; *P* = .02), Child-Pugh class B vs A (adjusted HR, 1.43; 95% CI, 1.13-2.25; *P* = .04) and C vs A (adjusted HR, 1.98; 95% CI, 1.18-3.31; *P* = .009), serum albumin (adjusted HR, 0.61; 95% CI, 0.43-0.86; *P* = .005), and newly developed HCC (adjusted HR, 1.68; 95% CI, 1.12-2.89; *P* = .01) remained significantly associated with mortality (Table 3). In addition, the GA/AA genotype and alcohol intake greater than 160 g per day (crude HR, 2.84; 95% CI, 1.89-4.27; *P* < .001) were significantly associated with an increased incidence of mortality compared with the GG genotype and an alcohol intake of 80 to 160 g per day (Figure 2C). High serum HBV DNA (≥5 log_10_ IU/mL) and the administration of antiviral therapy (crude HR, 1.64; 95% CI, 1.17-2.29; *P* = .003) were associated with an increased incidence of mortality compared with low serum HBV DNA levels (<5 log_10_ IU/mL) and the administration of antiviral therapy (Figure 2D). See additional findings in eAppendix 2 and eFigure 4 in the Supplement.

## Discussion

This cohort study showed that the 10-year cumulative incidences of HCC and mortality were significantly higher in patients with cirrhosis with concomitant HBV infection and alcoholism than in those with HBV infection alone or alcoholism alone before and after PSM. Furthermore, heavy alcohol intake and *ALDH2* rs671 polymorphism were significantly associated with increased risk of HCC development and mortality in patients with HBV-related cirrhosis. Our results are consistent with those of previous studies,^8,16,17,29,30^ which demonstrated that the synergistic effect of viral hepatitis infection and heavy alcohol intake aggravated the progression of HCC and mortality. It is important to closely follow-up and aggressively treat these patients with cirrhosis to decrease the incidence of HCC and mortality.

The *ALDH2* rs671 polymorphism is associated with the risk of HCC in patients with alcoholism with or without viral hepatitis^18,19,20,21^ and in patients without alcoholism.^22,23^ However, a meta-analysis^24^ showed that *ALDH2* rs671 polymorphism is not associated with HCC susceptibility in most East Asian patients with HBV or hepatitis C virus. Moreover, some studies^25,26,27^ also have demonstrated that the *ALDH2* rs671 polymorphism is not associated with HCC in East Asian patients. Our study revealed that the *ALDH2* rs671 polymorphism (GA/AA genotype) was significantly associated with the incidence and risk of HCC and mortality compared with the GG genotype in patients with cirrhosis with alcoholism regardless of their HBV infection status. In addition, the GA/AA genotype and an alcohol intake greater than 160 g per day were associated with an increased incidence of HCC and mortality compared with the GG genotype and an alcohol intake of 80 to 160 g per day. Thus, *ALDH2* rs671 polymorphism is an important factor associated with the risk of HCC and mortality in patients with cirrhosis with alcoholism, with or without HBV infection. Our results are consistent with previous studies^18,19,20,21^ showing that *ALDH2* rs671 polymorphism is associated with the risk of HCC in patients with alcoholism with or without viral hepatitis. To the best of our knowledge, our study is the first to report that heavy alcohol intake combined with *ALDH2* rs671 polymorphism is significantly associated with the risk of HCC and mortality in patients with alcoholism with cirrhosis after a 10-year, long-term follow-up. In addition, our study showed that *ALDH2* rs671 polymorphism is not significantly associated with HCC or mortality in patients with HBV-related cirrhosis without heavy alcoholism. Our results are consistent with previous studies^24,26,27^ showing that *ALDH2* rs671 polymorphism is not significantly associated with HCC but are not consistent with previous studies^18,19,20,21,22,23^ showing that *ALDH2* rs671 polymorphism is significantly associated with HCC. *ALDH2* rs671 polymorphism was associated with the risk of HCC and mortality in patients with alcoholism with or without viral hepatitis. However, whether *ALDH2* rs671 polymorphism is a risk factor for HCC in patients without alcoholism remains unclear and requires further investigation in a prospective and large cohort study.

The factors associated with HCC include viral factors, such as baseline serum HBV DNA and antiviral therapy, alcoholic factors, such as alcohol intake and abstinence, and genetic factors, such as *ALDH2* rs671 polymorphism. Viral factors, alcoholic factors, and genetic factors are important for HCC development. Moreover, the factors associated with mortality included abstinence, *ALDH2* rs671 polymorphism, factors related to the severity of cirrhosis, such as Child-Pugh class and serum albumin, and newly developed HCC in patients with cirrhosis with HBV infection and alcoholism. Alcoholism, genetic factors, the severity of disease, and tumor-related factors are important for mortality.

Our data showed that serum HBV DNA levels are a factor significantly associated with HCC risk and that antiviral therapy was associated with significantly reduced incidence of HCC in patients with cirrhosis with HBV infection and alcoholism. Our findings confirm that increased HBV DNA levels are associated with the progression of liver cirrhosis to HCC^8,11^ and that antiviral therapy is associated with reduced incidence of HCC in patients with HBV.^8,12,13,14,15^ Furthermore, our study showed that antiviral therapy is associated with reduced incidence of mortality in patients with cirrhosis with HBV infection and alcoholism in the univariable analyses. Therefore, aggressive antiviral therapy is associated with reduced incidence of HCC and mortality in patients with cirrhosis with HBV infection and alcoholism.

### Limitations

The study has limitations that should be addressed. First, the retrospective nature of this study might have resulted in selection bias. Therefore, we conducted a large, multicenter study. Second, the patients may not accurately report their alcohol intake during follow-up, which possibly affects the data regarding abstinence.

## Conclusions

Patients with cirrhosis with concomitant HBV infection and heavy alcoholism had significantly higher incidences of HCC and mortality than those with HBV infection alone or heavy alcoholism alone before and after PSM. Furthermore, heavy alcohol intake and *ALDH2* rs671 polymorphism were significantly associated with increased risk of HCC and mortality in patients with HBV-related cirrhosis. Patients with these risk factors should be monitored closely for HCC.

## Notes

Supplement. **eAppendix 1.** Supplemental Materials and Methods **eAppendix 2.** Supplemental Results **eTable 1.** Demographic Data Between Concomitant HBV Infection and Heavy Alcoholism and HBV Infection Alone After Propensity Score Matching **eTable 2.** Demographic Data Between Concomitant HBV Infection and Heavy Alcoholism and Alcoholism Alone After Propensity Score Matching **eTable 3.** Demographic Data Between HBV Infection Alone and Heavy Alcoholism Alone After Propensity Score Matching **eFigure 1.** Study Flowchart for the Inclusion of Participants **eFigure 2.** The Cumulative Incidences of HCC and Mortality After Propensity Score Matching **eFigure 3.** The Cumulative Incidences of HCC With Competing Risk Analysis **eFigure 4.** The Cumulative Incidences of HCC and Mortality in Compensated Cirrhotic Patients
