# Supplementary material for: Association of Heavy Alcohol Intake and ALDH2 rs671 Polymorphism With Hepatocellular Carcinoma and Mortality in Patients With Hepatitis B Virus–Related Cirrhosis
Source: JAMA Netw Open. 2022 Jul 25;5(7):e2223511. doi: 10.1001/jamanetworkopen.2022.23511 (PMC9315423; doi:10.1001/jamanetworkopen.2022.23511)
Supplement: Supplement. — eAppendix 1. Supplemental Materials and Methods eAppendix 2. Supplemental Results eTable 1. Demographic Data Between Concomitant HBV Infection and Heavy Alcoholism and HBV Infection Alone After Propensity Score Matching eTable 2. Demographic Data Between Concomitant HBV Infection and Heavy Alcoholism and Alcoholism Alone After Propensity Score Matching eTable 3. Demographic Data Between HBV Infection Alone and Heavy Alcoholism Alone After Propensity Score Matching eFigure 1. Study Flowchart for the Inclusion of Participants eFigure 2. The Cumulative Incidences of HCC and Mortality After Propensity Score Matching eFigure 3. The Cumulative Incidences of HCC With Competing Risk Analysis eFigure 4. The Cumulative Incidences of HCC and Mortality in Compensated Cirrhotic Patients [file jamanetwopen-e2223511-s001.pdf]

Supplemental Online Content

Tsai MC, Yang SS, Lin CC, et al. Association of heavy alcohol intake and *ALDH2* rs671 polymorphism with hepatocellular carcinoma and mortality in patients with hepatitis B virus–related cirrhosis. *JAMA Netw Open*. 2022;5(7):e2223511. doi:10.1001/jamanetworkopen.2022.23511

eAppendix 1. Supplemental Materials and Methods
eAppendix 2. Supplemental Results
eTable 1. Demographic Data Between Concomitant HBV Infection and Heavy Alcoholism and HBV Infection Alone After Propensity Score Matching
eTable 2. Demographic Data Between Concomitant HBV Infection and Heavy Alcoholism and Alcoholism Alone After Propensity Score Matching
eTable 3. Demographic Data Between HBV Infection Alone and Heavy Alcoholism Alone After Propensity Score Matching
eFigure 1. Study Flowchart for the Inclusion of Participants
eFigure 2. The Cumulative Incidences of HCC and Mortality After Propensity Score Matching
eFigure 3. The Cumulative Incidences of HCC With Competing Risk Analysis
eFigure 4. The Cumulative Incidences of HCC and Mortality in Compensated Cirrhotic Patients

This supplemental material has been provided by the authors to give readers additional information about their work.

© 2022 Tsai MC et al. *JAMA Network Open.*

eAppendix 1. Supplemental Materials and Methods

HBV marker assay

All HBV patients were tested for HBsAg, hepatitis B e antigen (HBeAg) and anti-HBe antibody, as well as serum HBV DNA with a lower detection limit of 15 IU/mL.

Treatments

Alcoholic patients were encouraged to abstain from alcohol. Abstinence was defined as abstaining from alcohol for more than 6 months during follow-up. Antiviral NUC therapy was administered if patients had one of serum HBV DNA ≥2000 IU/mL, total bilirubin ≥2 mg/dl, or prolonged prothrombin time >3 seconds in HBV-related cirrhotic patients according to National Health Insurance in Taiwan. Nine hundred and seventy-five patients received antiviral therapy and the mean time of antiviral therapy was 4.0 years. HCC treatments included surgical resection, radiofrequency ablation, transarterial chemoembolization, targeted therapy, immunotherapy, hepatic artery infusion chemotherapy, radiotherapy, and best supportive care.

© 2022 Tsai MC et al. *JAMA Network Open.*

eAppendix 2. Supplemental Results

Newly developed HCC and mortality in cirrhotic patients with Child-Pugh class A

Of the patients with compensated cirrhosis, 38 (22.1%) patients with concomitant HBV infection and alcoholism, 41 (12.9%) patients with HBV infection alone, and 12 (7.7%) patients with alcoholism alone were found to have newly developed HCC during follow-up. The 1-, 3-, 5-, and 10-year cumulative incidence rates of HCC were 6.5%, 17.3%, 21.1%, and 35.9%, respectively, for patients with concomitant HBV infection and alcoholism; 2.6%, 9.4%, 11%, and 22.8% for patients with HBV infection alone; and 0%, 3.8%, 3.8%, and 14.7% for patients with alcoholism alone (eFigure 4A). In Child-Pugh class A patients, the 10-year cumulative incidence rates of HCC were higher in patients with concomitant HBV infection and alcoholism than in those with HBV infection alone (HR= 2.58; 95% CI: 1.66-4.01, P<.001) or with alcoholism alone (HR= 4.12; 95% CI: 2.15-7.91, P<.001). For cirrhotic patients with Child-Pugh classes B and C, the cumulative incidence of HCC did not show significant differences.

Regarding mortality, 47 (27.3%) patients with concomitant HBV infection and alcoholism, 91 (28.7%) patients with HBV infection alone, and 36 (29.7%) patients with alcoholism alone experienced mortality during follow-up. The 1-, 3-, 5-, and 10-year cumulative incidence rates of mortality were 2.4%, 22.7%, 26.4%, and 47.3%, respectively, for patients with concomitant HBV infection and alcoholism; 1.9%, 12.9%, 27.2%, and 36.3% for patients with HBV infection alone; and 1.8%, 3.7%, 14.4%, and 45.1% for patients with alcoholism alone (eFigure 4B). The mortality rate was not significantly different among the three groups of Child-Pugh classes A, B, and C.

© 2022 Tsai MC et al. *JAMA Network Open.*

eTable 1. Demographic Data Between Concomitant HBV Infection and Heavy Alcoholism and HBV Infection Alone After Propensity Score Matching

**Characteristics**	**HBV+alcoholism (n =266)**	**HBV (n =796)**	**SMD**	***P* value**
Age, mean (SD), y	49.8 (10.1)	50.5 (10.4)	0.16	0.39
Gender, male	220 (82.7)	647 (81.3)	0.07	0.68
BMI (kg/m^2)	24.1 (3.5)	23.7 (3.4)	0.09	0.53
Alcohol intake, mean (SD), g/day	182 (80)	0 (0)		<.001
Alcohol duration, mean (SD), y	17.9 (6.9)	0 (0)		<.001
Abstinence	139 (52.6)	NA		NA
AST, mean (SD), U/L	138 (149)	152 (121)		0.07
ALT, mean (SD), U/L	62 (50)	64 (58)		0.76
Total bilirubin, mean (SD), mg/dL	3.4 (3.3)	4.0 (5.6)		0.11
Alk-P, mean (SD), IU/L	351 (226)	322 (180)		0.06
γ-GT, mean (SD), IU/L	319 (306)	331 (303)		0.25
Albumin, mean (SD), g/dL	3.3 (0.6)	3.3 (0.6)		0.97
Platelet count, mean (SD), x10^3/mL	106 (75)	120 (106)		0.13
INR, mean (SD)	1.3 (0.5)	1.3 (0.3)		0.78
α-fetoprotein, mean (SD), ng/mL	36 (111)	27 (95)		0.08
HBsAg-positive	266 (100)	796 (100)		0.99
HBeAg-positive	89 (33.4)	270 (33.9)		0.88
Baseline HBV DNA, mean (SD), log_{10} IU/mL	4.3 (2.2)	4.2 (2.3)		0.93
Baseline HBV DNA, ≥5 log_{10} IU/mL	111 (41.7)	313 (39.3)		0.41
Antiviral viral therapy -positive	228 (85.7)	667 (83.8)		0.15
Child-Pugh class				
A	111 (41.7)	317 (39.8)	0.18	0.27
B	84 (31.6)	316 (39.7)		
C	71 (26.7)	163 (20.5)		

Data shown as number (%) or mean (SD); SMD: Standardized mean difference; BMI: Body mass index; AST: Aspartate aminotransferase; ALT: Alanine aminotransferase; Alk-P: Alkaline phosphatase; γ -GT: γ-glutamyltransferase; INR: international normalized ratio; HBsAg: Hepatitis B Surface Antigen; HBeAg: Hepatitis B e Antigen; HBV: Hepatitis B Virus; NA: data not available;

© 2022 Tsai MC et al. *JAMA Network Open.*

eTable 2. Demographic Data Between Concomitant HBV Infection and Heavy Alcoholism and Alcoholism Alone After Propensity Score Matching

**Characteristics**	**HBV+alcoholism (n =280)**	**Alcoholism (n =280)**	**SMD**	***P* value**
Age, mean (SD), y	49.7 (9.9)	50.1 (10.1)	0.06	0.71
Gender, male	238 (85.0)	235 (83.9)	0.03	0.83
BMI (kg/m^2)	24.2 (3.8)	23.9 (3.9)	0.07	0.66
Alcohol intake, mean (SD), g/day	186 (81)	178 (91)		0.51
Alcohol duration, mean (SD), y	18.1 (6.3)	17.9 (6.6)		0.87
Abstinence	148 (52.9)	163 (58.2)		0.18
AST, mean (SD), U/L	136 (139)	147 (125)		0.07
ALT, mean (SD), U/L	64 (51)	60 (51)		0.42
Total bilirubin, mean (SD), mg/dL	3.1 (3.3)	3.7 (5.4)		0.08
Alk-P, mean (SD), IU/L	378 (215)	370 (202)		0.61
γ-GT, mean (SD), IU/L	308 (296)	337 (285)		0.17
Albumin, mean (SD), g/dL	3.2 (0.6)	3.2 (0.7)		0.95
Platelet count, mean (SD), x10^3/mL	103 (92)	111 (115)		0.07
INR, mean (SD)	1.3 (0.4)	1.3 (0.3)		0.68
α-fetoprotein, mean (SD), ng/mL	38 (113)	27 (101)		0.17
HBsAg-positive	280 (100)	0 (0)		<.001
HBeAg-positive	76 (27.1)	NA		NA
Baseline HBV DNA, mean (SD), $\log_{10}$ IU/mL	4.4 (2.1)	NA		NA
Baseline HBV DNA, ≥5 $\log_{10}$ IU/mL	118 (42.1)	NA		NA
Antiviral viral therapy -positive	258 (92.1)	NA		NA
Child-Pugh class				
A	133 (47.5)	127 (45.7)	0.09	0.57
B	74 (26.4)	78 (27.6)		
C	73 (26.1)	75 (26.7)		

Data shown as number (%) or mean (SD); SMD: Standardized mean difference; BMI: Body mass index; AST: Aspartate aminotransferase; ALT: Alanine aminotransferase; Alk-P: Alkaline phosphatase; γ -GT: γ-glutamyltransferase; INR: international normalized ratio; HBsAg: Hepatitis B Surface Antigen; HBeAg: Hepatitis B e Antigen; HBV: Hepatitis B Virus; NA: data not available;

© 2022 Tsai MC et al. *JAMA Network Open.*

eTable 3. Demographic Data Between HBV Infection Alone and Heavy Alcoholism Alone After Propensity Score Matching

Characteristics	Alcoholism (n =266)	HBV (n =796)	SMD	*P* value
Age, mean (SD), y	50.7 (10.1)	50.5 (10.4)	0.01	0.92
Gender, male	218 (81.9)	647 (81.3)	0.03	0.81
BMI (kg/m^2)	23.8 (3.6)	23.7 (3.4)	0.04	0.79
Alcohol intake, mean (SD), g/day	177 (93)	0 (0)		<.001
Alcohol duration, mean (SD), y	17.3 (6.2)	0 (0)		<.001
Abstinence	163 (61.3)	NA		NA
AST, mean (SD), U/L	145 (116)	152 (121)		0.46
ALT, mean (SD), U/L	59 (50)	64 (58)		0.59
Total bilirubin, mean (SD), mg/dL	3.8 (5.5)	4.0 (5.6)		0.77
Alk-P, mean (SD), IU/L	362 (209)	322 (180)		0.06
γ-GT, mean (SD), IU/L	343 (257)	331 (303)		0.29
Albumin, mean (SD), g/dL	3.6 (0.6)	3.3 (0.6)		0.96
Platelet count, mean (SD), x10^3/mL	112 (110)	120 (106)		0.23
INR, mean (SD)	1.3 (0.3)	1.3 (0.3)		0.68
α-fetoprotein, mean (SD), ng/mL	22 (69)	27 (95)		0.27
HBsAg-positive	0 (0)	796 (100)		<.001
HBeAg-positive	NA	270 (33.9)		NA
Baseline HBV DNA, mean (SD), $\log_{10}$ IU/mL	NA	4.2 (2.3)		NA
Baseline HBV DNA, ≥5 $\log_{10}$ IU/mL	NA	313 (39.3)		NA
Antiviral viral therapy -positive	NA	667 (83.8)		NA
Child-Pugh class				
A	110 (41.3)	317 (39.8)	0.11	0.65
B	106 (39.9)	316 (39.7)		
C	50 (18.8)	163 (20.5)		

Data shown as number (%) or mean (SD); SMD: Standardized mean difference; BMI: Body mass index;

AST: Aspartate aminotransferase; ALT: Alanine aminotransferase; Alk-P: Alkaline phosphatase;

γ -GT: γ-glutamyltransferase; INR: international normalized ratio; HBsAg: Hepatitis B Surface Antigen;

HBeAg: Hepatitis B e Antigen; HBV: Hepatitis B Virus; NA: data not available;

© 2022 Tsai MC et al. *JAMA Network Open.*

eFigure 1. Study Flowchart for the Inclusion of Participants

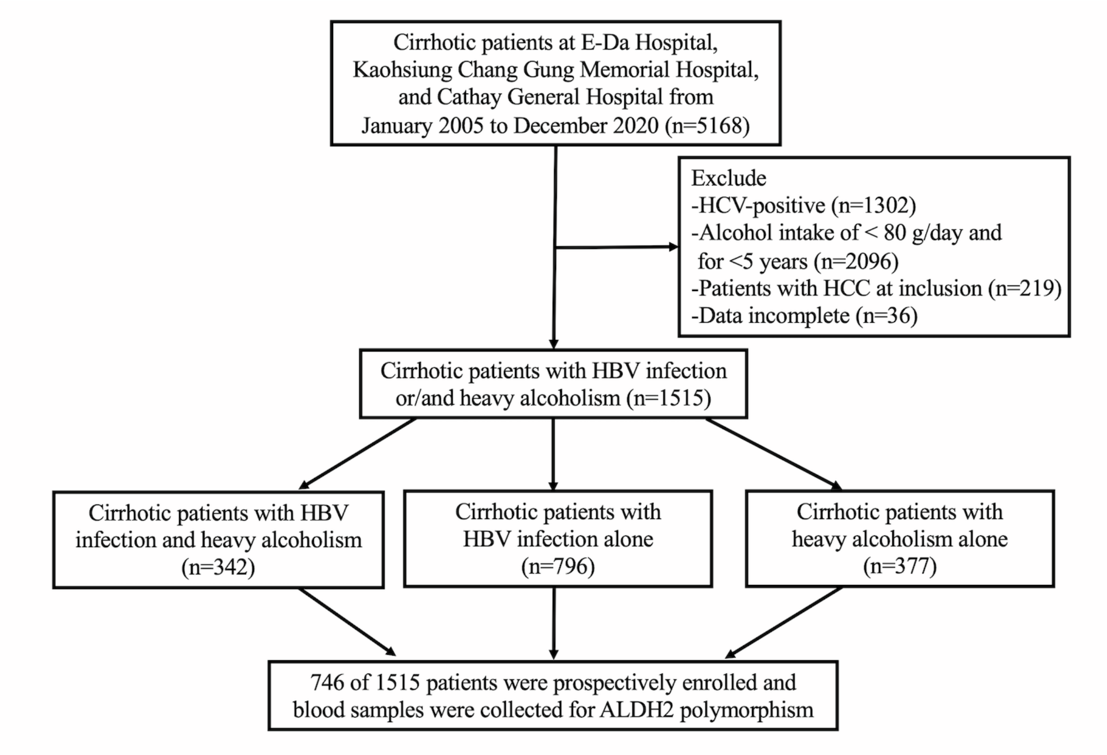

© 2022 Tsai MC et al. *JAMA Network Open.*

eFigure 2. The Cumulative Incidences of HCC and Mortality After Propensity Score Matching

The cumulative incidences of HCC (A-B) and mortality (D-E) were higher in cirrhotic patients with concomitant HBV infection and alcoholism than in those with HBV infection alone or alcoholism alone. The cumulative incidences of HCC (C) and mortality (F) were not significant difference between patients with HBV infection alone and those with alcoholism alone.

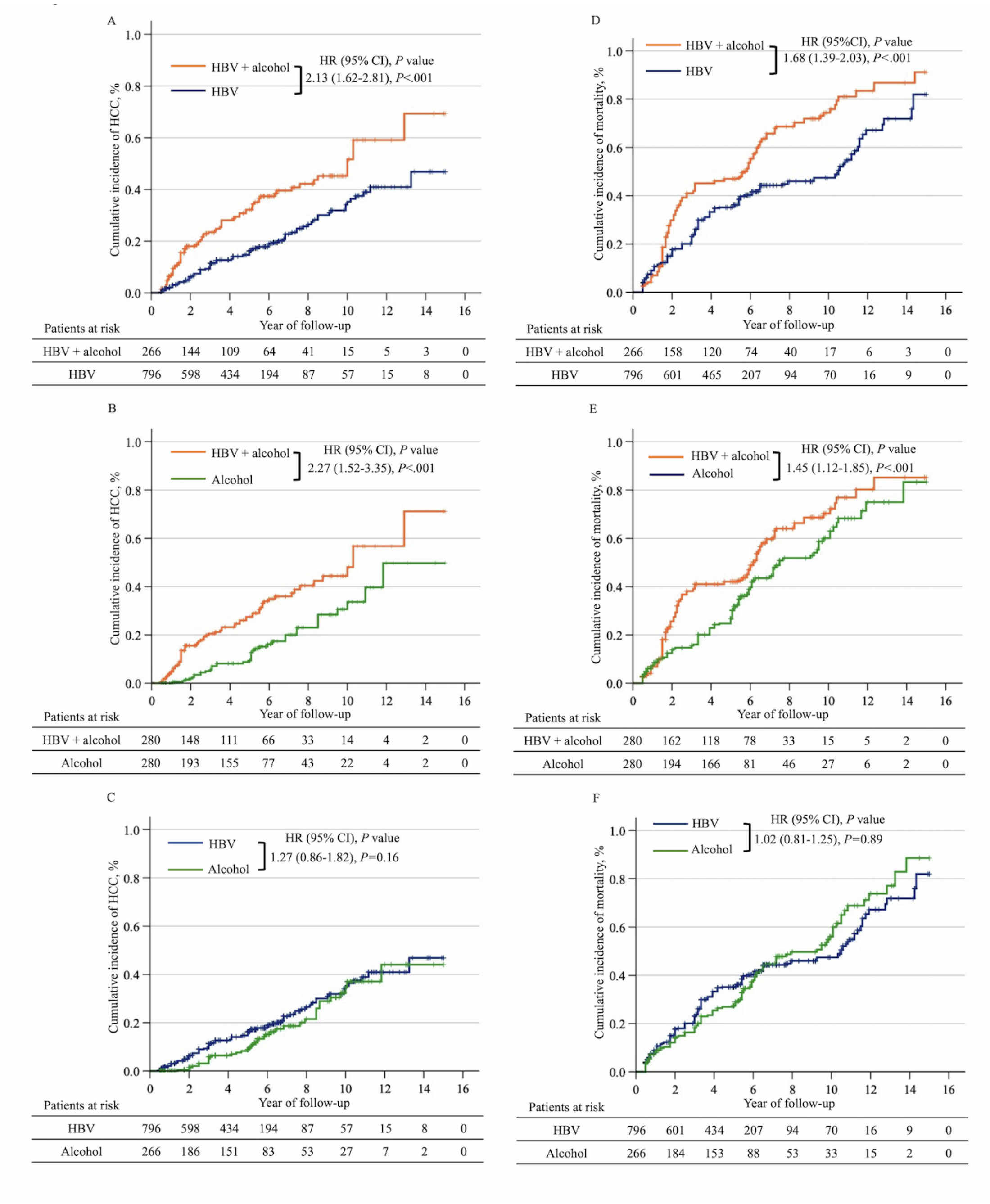

© 2022 Tsai MC et al. *JAMA Network Open.*

eFigure 3. The Cumulative Incidences of HCC With Competing Risk Analysis

The cumulative incidences of HCC (A-B) were higher in cirrhotic patients with concomitant HBV infection and alcoholism than in those with HBV infection alone or alcoholism alone. The cumulative incidences of HCC (C) were not significant difference between patients with HBV infection alone and those with alcoholism alone.

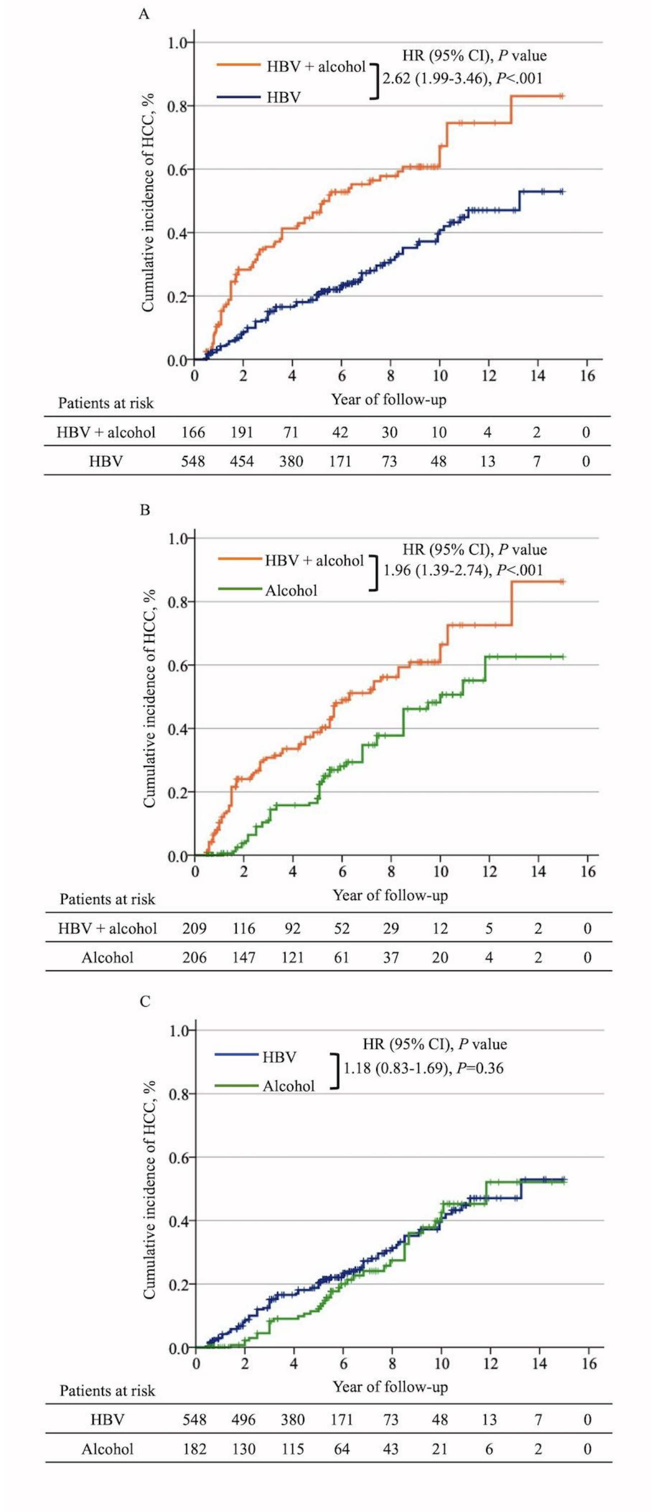

© 2022 Tsai MC et al. *JAMA Network Open.*

eFigure 4. The Cumulative Incidences of HCC and Mortality in Compensated Cirrhotic Patients

The cumulative incidences of HCC (A) and mortality (B) were higher in compensated cirrhotic patients with concomitant HBV infection and alcoholism than in those with HBV infection alone or alcoholism alone.

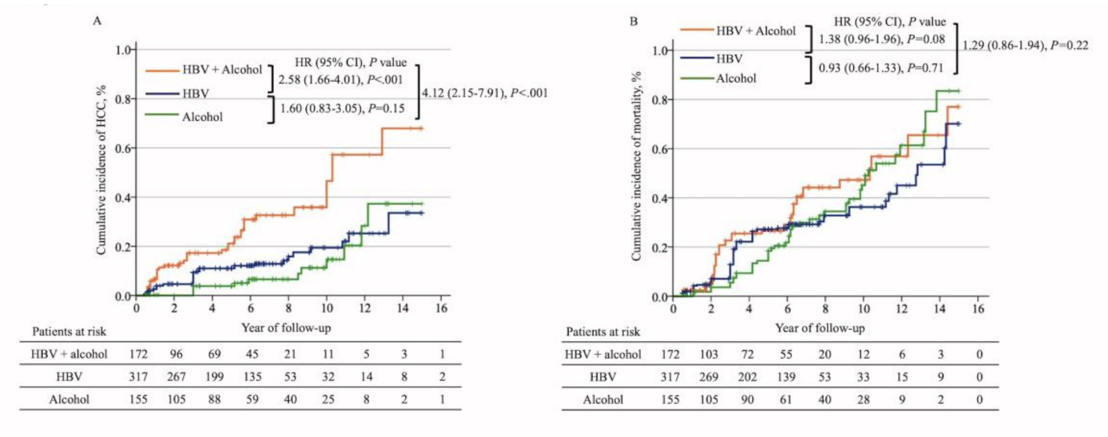

© 2022 Tsai MC et al. *JAMA Network Open.*
